# The clinical value of autologous platelet-rich plasma extraction and injection as an adjunct to urethroplasty in the treatment of penile hypospadias in children

**DOI:** 10.3389/fped.2025.1470092

**Published:** 2025-04-30

**Authors:** Xianhui Shang, Zhendong Zhang, Kaiyi Mao, Hongyanng Tang, Guangxu Zhou, Yuchen Mao, Yingbo Li, Zhen Luo, Peng Zhao, Cao Wang, Hong Ma

**Affiliations:** ^1^ Department of Pediatric Surgery, Affiliated Hospital of Zunyi Medical University, Zunyi, China; ^2^ Department of Pediatric Surgery, Guizhou Children's Hospital, Zunyi, China; ^3^ Department of Gynaecology, Affiliated Hospital of Zunyi Medical University, Zunyi, China

**Keywords:** platelet-rich plasma, penile hypospadias, urethroplasty, wound healing, postoperative complications, pediatric surgery

## Abstract

**Objective:**

To evaluate the clinical efficacy and safety of autologous platelet-rich plasma (PRP) as an adjunctive treatment for penile hypospadias repair in children, and to explore its independent role in reducing postoperative complications.

**Methods:**

We retrospectively analyzed clinical data from 103 pediatric patients undergoing penile hypospadias repair between December 2019 and December 2021 at the Affiliated Hospital of Zunyi Medical University. All patients received standard penile straightening and tubularized incised plate (TIP) urethroplasty. Patients in the study group (*n* = 53) additionally received intraoperative autologous PRP injections, whereas the control group (*n* = 50) did not. Outcomes analyzed included operation time, postoperative ambulation time, pain scores, length of hospital stay, incision infection rates at postoperative day 7, surgical success rates, and incidence of complications within 2 years postoperatively. Statistical analyses incorporated 95% confidence intervals (CIs), effect sizes (Cohen's d and relative risk, RR), and multivariate logistic regression analyses adjusting for potential confounders such as patient age and hypospadias severity.

**Results:**

No significant differences were observed between groups regarding operation time, postoperative ambulation time, or length of hospital stay (*p* > 0.05). Patients in the PRP group experienced significantly reduced postoperative pain (mean difference −2.14; 95% CI: −2.46 to −1.81; *p* < 0.001; Cohen's d = 2.35) and notably lower incision infection rates on postoperative day 7 (RR = 0.13; 95% CI: 0.03–0.60; *p* = 0.006). Surgical success rates were significantly higher in the PRP group compared to controls (94.3% vs. 72.0%; RR = 1.31; 95% CI: 1.09–1.58; *p* = 0.002). Multivariate logistic regression analysis confirmed that PRP injection remained independently associated with a significant reduction in postoperative complications after adjusting for age and severity of hypospadias (adjusted OR = 0.14; 95% CI: 0.04–0.52; *p* = 0.003).

**Conclusion:**

Adjunctive autologous PRP treatment in pediatric penile hypospadias repair effectively alleviates postoperative pain, enhances wound healing, significantly reduces short-term complications, and improves surgical success rates. Future randomized, multicenter trials with extended follow-up periods are required to further evaluate long-term outcomes and to compare PRP efficacy directly with other biomaterials used in urethroplasty.

## Introduction

Hypospadias is one of the most common congenital abnormalities affecting the male genitourinary system, with a global incidence of approximately 0.3% that has gradually increased in recent years (1). In children affected by hypospadias, the abnormal positioning of the urethral meatus not only impacts urinary function but may also adversely affect sexual function later in adulthood (2). Surgery is the only definitive treatment for hypospadias; however, clinicians continue to face significant challenges due to delayed wound healing and a high incidence of postoperative complications (3).

In recent years, platelet-rich plasma (PRP), a novel biologic product derived by centrifugation of autologous venous blood, has attracted growing attention. PRP is characterized by high concentrations of platelets, plasma, and various growth factors, including vascular endothelial growth factor (VEGF), platelet-derived growth factor (PDGF), and transforming growth factor-beta (TGF-β). These growth factors have been shown to accelerate early wound closure, enhance collagen synthesis and tissue regeneration, and exhibit antimicrobial effects (4, 5). PRP has been widely applied in various clinical specialties, including oral and maxillofacial surgery, plastic and aesthetic surgery, dermatology, and orthopedics, demonstrating significant efficacy in promoting wound healing and tissue regeneration.

Currently, the majority of postoperative complications of hypospadias repair, such as urethral fistula, urethral stricture, and infections, are attributed to surgical technical issues or impaired wound healing (3). Clinical studies have suggested that the multiple growth factors present in PRP can significantly accelerate postoperative wound healing, thereby reducing complication rates (6).

Therefore, this retrospective study aims to evaluate the clinical efficacy of PRP as an adjunctive treatment in reducing postoperative complications following penile hypospadias repair in children. We performed multivariate analyses to adjust for potential confounding factors and further clarify the independent therapeutic effects of PRP. This work aims to provide a reliable basis for future randomized controlled trials.

## Methods

### Sample size estimation

In this study, we observed the differences in the efficacy of different modalities in the treatment of pediatric hypospadias, and reviewed the literature to know that the incidence of postoperative complications in the study group and the control group were 5.22% and 32.16%, respectively. Based on the sample size formula $n=\frac{2\overset{-}{p}\times \overset{-}{q}\;{\left(Z_{\alpha }+Z_{\beta }\right)}^{2}}{{\left(\;p_{1}-p_{2}\right)}^{2}}$, in this study, *α* = 0.05, *β* = 0.1, Z*α* = 1.96, Z*β* = 1.28, with $\bar{q}$ being the mean of p1 and p2, and $\bar{q}$ being the mean of 1-p1 and 1-p2, and p1 = 0.0522, p2 = 0.3216. In this study, p1 = 0.0522, p2 = 0.3216, $n=\frac{2\times 0.1891\times 0.8109\times {\left(1.96+1.28\right)}^{2}}{{\left(0.0522-0.3216\right)}^{2}}$ ≈44, so each group needs to include at least 44 observation subjects, and the study group and the control group in this study included 53 and 50 study subjects, respectively.

### Inclusion and exclusion criteria

We retrospectively analyzed clinical data from 103 children treated for penile hypospadias at the Affiliated Hospital of Zunyi Medical University from December 2019 to December 2021. The inclusion criteria were as follows: (1) patients younger than 14 years diagnosed with penile hypospadias according to the Chinese Expert Consensus on the Diagnosis and Treatment of Hypospadias (2016) (7); (2) patients undergoing tubularized incised plate (TIP) urethroplasty, with routine catheter retention for 10 days postoperatively; (3) patients with complete postoperative follow-up data. Exclusion criteria included: (1) concurrent hematological or immunodeficiency diseases; (2) secondary surgical repairs due to previous surgical failure or complications; (3) incomplete follow-up data or lost to follow-up.

### Surgical methods

In the control group, all children underwent standard TIP urethroplasty performed by a single experienced surgeon. Briefly, after successful anesthesia, patients were placed in a supine position. The glans penis was suspended and stabilized with sutures, and a urethral catheter was inserted. A U-shaped incision was made bilaterally at the urethral meatus and extended to the tip of the glans penis groove. The penile deep fascia was degloved to the penile root, and excessive tissue on the ventral penile aspect was completely excised. Most penile curvature was corrected by this procedure alone, although severe cases required dorsal tunica albuginea folding sutures for straightening. Subsequently, the urethral plate edges were sutured using 6-0 absorbable sutures to form a neo-urethra. The dorsal dartos flap was then transferred to the ventral side to cover the proximal neo-urethra and create the neo-glans.

**Figure 1:**
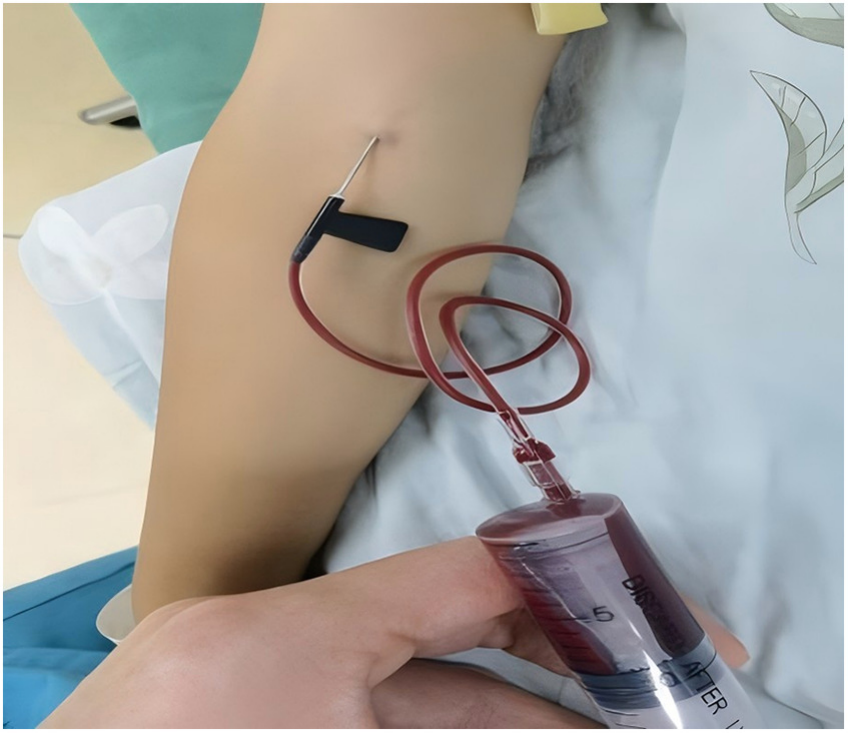
Collection of venous blood from the patient.

**Figure 2:**
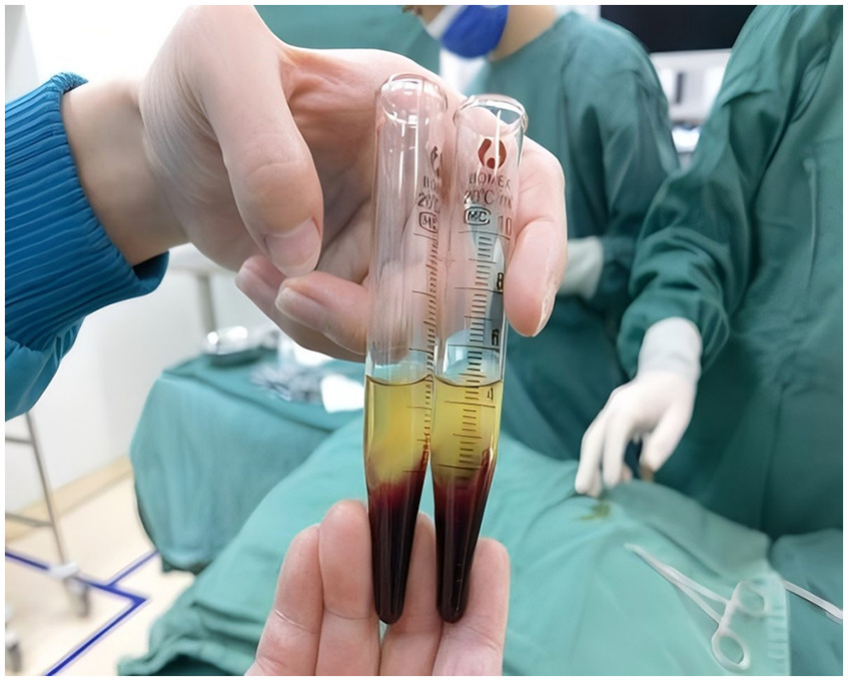
Preparation of platelet-rich plasma (PRP) by centrifugation.

**Figure 3:**
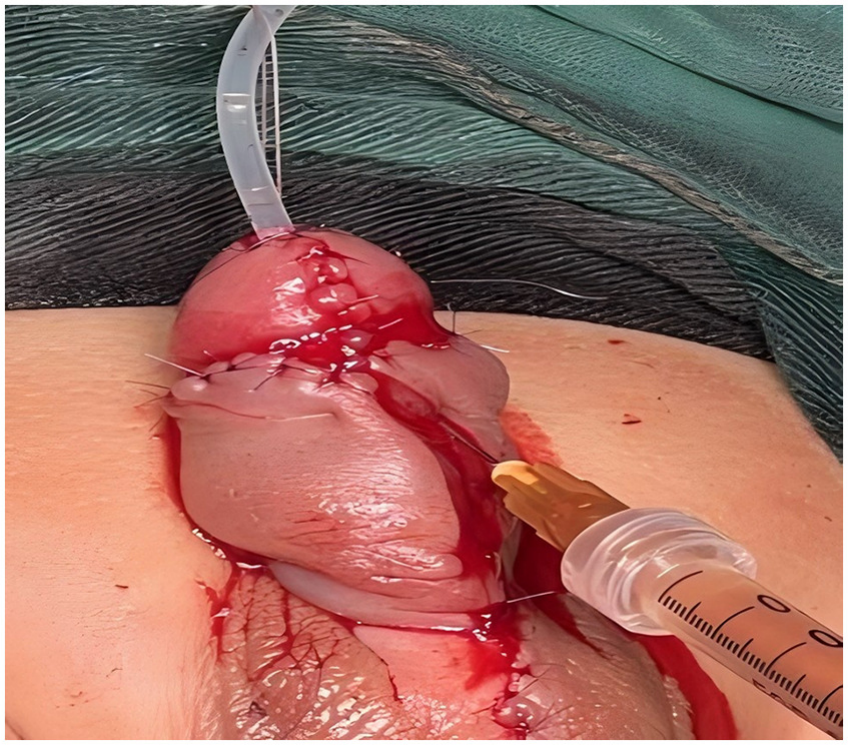
Injection of platelet-rich plasma (PRP).

In the study group, patients received autologous platelet-rich plasma (PRP) injections as an adjunct to urethroplasty, which was otherwise performed identically to the control group. PRP preparation involved collecting 10 ml of venous blood prior to surgery, which was subjected to initial centrifugation (Figure 1). The supernatant and the plasma approximately 3 mm below the plasma interface were then carefully harvested and subjected to a second centrifugation. Subsequently, three-quarters of the supernatant were discarded, and the remaining liquid and precipitate were retained as PRP (Figure 2). Following penile straightening and urethroplasty, a total of 3 ml of PRP was slowly and evenly injected subcutaneously along both sides of the urethral plate, between the skin and dorsal dartos flap, using a syringe (Figure 3).

Postoperatively, both groups received identical wound care, including aseptic Vaseline gauze dressings and elastic bandages, routine catheterization, and antibiotic prophylaxis. Follow-up was conducted for 2 years. Two patients were lost to follow-up in the study group, and three patients were lost to follow-up in the control group.

### Outcome measures

The following outcome measures were evaluated:

1. Surgical outcomes: operation time, postoperative ambulation time, pain scores [using the validated Chinese version of the Child Pain Behavior Scale (8)], and duration of hospital stay.
2. Surgical success rate at 2 years post-surgery, defined by unobstructed urination without complications such as urethral stricture, urethral fistula, diverticulum, or wound dehiscence (9).
3. Wound healing at postoperative day 7, classified into Grade A (optimal healing without adverse reactions), Grade B (mild inflammatory reactions such as redness or exudation without purulence), and Grade C (purulent exudate requiring clinical intervention) (10).
4. Incidence of postoperative complications including urethral stricture, urethral fistula, urethral diverticulum, wound dehiscence, and infection within 2 years postoperatively.

### Statistical analysis

Statistical analyses were performed using SPSS software version 29.0. Normally distributed continuous variables were expressed as mean ± standard deviation (mean ± SD), and comparisons between groups were conducted using independent-samples *t*-tests, accompanied by calculation of 95% confidence intervals (95% CI) and effect sizes (Cohen's d). Categorical variables were presented as numbers and percentages (*n*, %) and analyzed by chi-square tests, with relative risk (RR) and corresponding 95% CI calculated. Multivariate logistic regression analysis was further applied to adjust for potential confounders, such as age and hypospadias severity, to clarify the independent therapeutic effect of PRP treatment. A two-tailed *p*-value of less than 0.05 was considered statistically significant.

## Results

### Comparison of surgical outcomes

No significant differences were found between the two groups in operation time, postoperative ambulation time, or length of hospital stay (all *p* > 0.05). However, the PRP group demonstrated significantly lower postoperative pain scores compared to the control group, with a mean difference of −2.14 (95% CI: −2.46 to −1.81; Cohen's d = 2.35, *p* < 0.001) (Table 1).

**Table 1:** Comparison of surgical outcomes between the two groups (mean ± SD).

Group	*n*	Operation time (h)	Postoperative ambulation Time (days)	Pain score (points)	Hospital stay (days)
PRP group	53	1.47 ± 0.53	2.31 ± 0.65	2.14 ± 0.53	4.37 ± 3.28
Control group	50	1.39 ± 0.61	2.38 ± 0.72	4.28 ± 1.17	5.29 ± 3.13
t value	–	0.702	0.511	12.005	1.430
*p* value	–	0.484	0.611	<0.001	0.156
Cohen's d	–	0.14	0.10	2.35	0.29
95% CI	–	−0.14 to 0.30	−0.20 to 0.34	−2.46 to −1.81	−2.18 to 0.34

### Incision healing status at postoperative day 7

At postoperative day 7, the PRP group exhibited significantly better incision healing (Grade A) and fewer incision infections (Grade C) than the control group (RR = 0.13, 95% CI: 0.03–0.60, *p* = 0.006) (Table 2).

**Table 2:** Comparison of incision healing status at postoperative day 7 [*n* (%)].

Group	*n*	Grade A (optimal healing)	Grade B (mild reaction)	Grade C (infection)
PRP group	53	50 (94.34%)	2 (3.77%)	1 (1.89%)
Control group	50	36 (72.00%)	7 (14.00%)	7 (14.00%)
*χ*^2^ value	–	9.160	3.326	4.884
*p* value	–	0.002	0.068	0.027
RR (95% CI)^a^	–	1.31 (1.09–1.58)	0.27 (0.06–1.28)	0.13 (0.03–0.60)

^a^ RR calculated using control group as reference.

### Surgical success rates and complications at 2-year follow-up

At 2-year follow-up, the surgical success rate in the PRP group was significantly higher compared to the control group (94.33% vs. 72.00%; RR = 1.31, 95% CI: 1.09–1.58, *p* = 0.002). The overall complication rate in the PRP group was significantly lower than that in the control group (5.66% vs. 28.00%; RR = 0.20, 95% CI: 0.06–0.66, *p* = 0.004) (Table 3).

**Table 3:** Surgical success rates and postoperative complications at 2 years [*n* (%)].

Group	*n*	Success rate	Stricture	Diverticulum	Infection	Fistula	Dehiscence	Total complications
PRP group	53	50 (94.33%)	1 (1.89%)	1 (1.89%)	1 (1.89%)	0 (0%)	0 (0%)	3 (5.66%)
Control group	50	36 (72.00%)	1 (2.00%)	1 (2.00%)	5 (10.00%)	6 (12.00%)	1 (2.00%)	14 (28.00%)
χ^2^ value	–	9.160	0.002	0.002	2.983	6.733	1.073	9.105
*p* value	–	0.002	0.963	0.963	0.084	0.009	0.300	0.004
RR (95% CI)^a^	–	1.31 (1.09–1.58)	0.94 (0.06–14.71)	0.94 (0.06–14.71)	0.19 (0.02–1.60)	0.07 (0.004–1.14)	0.31 (0.01–7.46)	0.20 (0.06–0.66)

^a^ RR calculated using control group as reference.

### Multivariate analysis adjusting for age and hypospadias severity

Multivariate logistic regression analysis, adjusted for age and severity of hypospadias, demonstrated that PRP injection was independently associated with significantly reduced risk of postoperative complications (adjusted OR = 0.14, 95% CI: 0.04–0.52, *p* = 0.003) (Table 4).

**Table 4:** Multivariate logistic regression analysis of postoperative complication risk.

Variable	Adjusted OR	95% CI	*p* value
Age	1.08	0.89–1.32	0.427
Severity of hypospadias	2.15	0.85–5.45	0.105
PRP treatment	0.14	0.04–0.52	0.003

^a^ OR calculated using control (no PRP treatment) as reference.

In summary, adjunctive PRP injection significantly reduced postoperative pain, improved incision healing, increased surgical success, and remained independently associated with decreased complication rates after adjustment for confounding factors.

## Discussion

Our study demonstrated that adjunctive autologous platelet-rich plasma (PRP) effectively promotes wound healing, significantly reduces postoperative complications, and alleviates postoperative pain following pediatric penile hypospadias repair. Notably, there were no statistically significant differences between the PRP and control groups regarding operative time, postoperative ambulation time, or hospital stay, indicating that PRP application did not impose additional surgical burden and affirming its clinical safety and feasibility. This finding aligns with previous reports indicating that PRP does not prolong surgical procedures.

In recent years, clinical research has increasingly focused on accelerating wound healing in pediatric surgical patients (11, 12). The therapeutic mechanism of PRP is primarily attributed to the concentrated release of multiple growth factors from platelets, including insulin-like growth factor (IGF), vascular endothelial growth factor (VEGF), transforming growth factor-beta (TGF-β), platelet-derived growth factor (PDGF), epidermal growth factor (EGF), and basic fibroblast growth factor (bFGF). Among these, VEGF, TGF-β, and PDGF play crucial roles. VEGF is a powerful stimulator of angiogenesis, while TGF-β enhances collagen synthesis and deposition, regulating cellular proliferation, division, and apoptosis. PDGF, predominantly stored in platelet granules, has chemotactic effects on macrophages and fibroblasts, enhancing early cellular activity and promoting deposition of fibronectin and glycosaminoglycan, thus accelerating wound healing and tissue regeneration (13).

PRP, prepared through centrifugation of autologous venous blood, contains high concentrations of platelets, leukocytes, and fibrin. Upon activation, PRP releases these growth factors, effectively promoting tissue regeneration and providing antibacterial protection (14). Ding L et al. demonstrated that PRP effectively alleviates pain and accelerates wound healing in chronic refractory wounds among elderly patients (15). Similarly, our study found significantly lower postoperative pain scores and reduced surgical site infections in the PRP group, consistent with previous findings that PRP promotes tissue regeneration and pain relief (16, 17).

Common complications following hypospadias repair include urethral fissures, fistulas, and strictures, which often result from impaired wound healing, localized inflammation, and associated pain (18). Our study demonstrated a notably lower incidence of postoperative complications in the PRP group, suggesting that PRP effectively reduces complications and improves surgical outcomes. This reduction can primarily be attributed to the anti-inflammatory and antibacterial properties of growth factors released by PRP, which alleviate local pain and enhance wound healing (19–21).

Currently, adjunctive biomaterials commonly used in urethroplasty include fibrin sealants and collagen matrices. Fibrin sealants enhance tissue adhesion, potentially reducing postoperative fistulas and leakage, but lack the biologically active growth factors necessary for active tissue regeneration. Collagen matrices, while providing structural support, similarly lack active compounds to stimulate wound healing and provide antimicrobial protection (22). Compared to these materials, PRP actively releases growth factors, directly enhancing tissue repair and reducing infection risk. Prospective randomized controlled trials comparing PRP directly with fibrin sealants and collagen matrices are thus warranted to clarify PRP's relative advantages.

Moreover, the 2-year follow-up period in our study demonstrated significant clinical advantages, particularly regarding reduced short-term complications such as fistulas and infections. However, a 2-year follow-up may not be sufficient for a comprehensive assessment of potential late-onset complications, including urethral strictures, persistent penile curvature, sexual dysfunction in adolescence or adulthood, and long-term patient satisfaction. Therefore, extending follow-up into adolescence or adulthood is necessary for thoroughly evaluating the long-term effectiveness of PRP.

The main limitations of our study include its retrospective nature, which inherently introduces selection and information biases, as well as the single-center and single-surgeon setting. Although this approach ensured surgical consistency, it limits the generalizability and external validity of our findings. Despite retrospective studies like Di Mitri et al. providing strong preliminary evidence supporting PRP effectiveness in specific pediatric surgical contexts, larger multicenter clinical trials remain essential (22). Hence, future multicenter, randomized controlled trials are necessary to confirm the clinical reliability and widespread applicability of PRP treatment.

In conclusion, PRP as an adjunct to pediatric hypospadias repair demonstrated clear short-term benefits in promoting wound healing, alleviating postoperative pain, and significantly reducing postoperative complications. However, given the relatively short follow-up period and limitations inherent to the study design, longer-term follow-up studies and direct comparisons with existing biomaterials are needed to definitively establish the long-term safety and efficacy of PRP in pediatric hypospadias repair.

## Data Availability

The original contributions presented in the study are included in the article/Supplementary Material, further inquiries can be directed to the corresponding author.
